# Association of S100B polymorphisms and serum S100B with risk of systemic lupus erythematous in a Chinese population

**DOI:** 10.1590/1678-4685-GMB-2017-0354

**Published:** 2019-07-01

**Authors:** Yulan Lu, Huatuo Huang, Chunhong Liu, Yonglong Zeng, Rong Wang, Chunfang Wang, Yesheng Wei, Yan Lan

**Affiliations:** 1 Department of Clinical Laboratory, Affiliated Hospital of Youjiang Medical University for Nationalities, No.18 Zhongshan Road II, Baise 533000, Guangxi, China; 2 Department of Dermatology, Affiliated Hospital of Youjiang Medical University for Nationalities, Guangxi, China

**Keywords:** S100B, polymorphisms, serum levels, SLE, neurologic disorder

## Abstract

The aim of this study was to investigate whether the S100B polymorphisms are associated with systemic lupus erythematous (SLE) in a Chinese population. A total of 313 SLE patients and 396 control subjects were enrolled in the present study. The genotypes of three SNPs (rs9722, rs881827 and rs1051169) in S100B gene were detected by single base extension polymerase chain reaction (SBE-PCR). Serum S100B levels were determined by enzyme-linked immunosorbent assay (ELISA). Rs1051169 was associated with an increased risk of SLE (C vs. G: adjusted OR=1.46, 95% CI, 1.18-1.80, *p*=0.001; CC vs. GG: adjusted OR=1.99, 95% CI, 1.32-3.02, *p*=0.001; CC+GC vs. GG: adjusted OR=1.54, 95% CI, 1.13-2.11, *p*=0.007; CC vs. GC+GG: adjusted OR=1.67, 95% CI, 1.16-2.42, *p*=0.006). Haplotype analysis showed that the G-G-C haplotype was associated with an increased risk of SLE (OR=1.50, 95% CI, 1.14-1.98, *p*=0.004). Stratified analyses showed that the rs1051169 polymorphism was associated with an increased risk of neurologic disorder in SLE patients (C vs. G: OR=1.78, 95% CI, 1.22-2.59, *p*=0.003; GC vs. GG: OR=2.33, 95% CI, 1.14-4.77, P=0.019; CC vs. GG: OR=3.02, 95% CI, 1.39-6.53, *p*=0.004; CC+GC vs. GG: OR=2.57, 95% CI=1.31-5.04, *p*=0.005). In addition, SLE patients with neurologic disorder carrying the rs1051169 GC/CC genotypes present a higher serum S100B levels compared with that carrying the GG genotype (*p* < 0.05). Our results indicate that the rs1051169 polymorphism may be involved in the pathogenesis of SLE.

## Introduction

Systemic lupus erythematous (SLE) is a common systemic autoimmune disease characterized by autoantibody production and immune complex deposition, resulting in damage to multiple tissues and organs, as well as physiological function impairment (Aringer, *et al.*, 2016). Most of the SLE patients will develop various symptoms, such as malar rash, arthritis, nephritis, and neurologic disorders (Wada, *et al.*, 2017). In China, the prevalence of SLE is 0.03% and it has come to be a heavy burden on family and society (Li, *et al.*, 2012; Holloway, *et al.*, 2014). It represents a primary challenge to health care and is considered as a major Chinese health concern. Yet to date, the exact pathogenic mechanism of SLE has not been fully elucidated. Several risk factors have been identified to contribute to the pathogenesis of SLE, such as genetic, environmental, infection, and hormonal factors. Among the well-known predisposing factors, genetic factors seem to play a key role in the susceptibility (Alarcon-Segovia, *et al.*, 2005; Tiffin, *et al.*, 2013; Ulff-Moller, *et al.*, 2017).

As is known, many cytokines are involved in the pathogenesis of autoimmune diseases. S100B is a member of the S100 family and primarily secreted by astrocytes, but to a lesser extent it is also produced by other cell types, such as dendritic cell, macrophages, monocytes, and T cells (Donato, *et al.*, 2009, 2013; Miki, *et al.*, 2013). S100B is thought to have intracellular and extracellular roles in the regulation of many diverse processes, such as cell growth and motility, cell-cycle regulation, transcription, differentiation, and Ca^2+^ homeostasis (Yardan, *et al.*, 2011). In addition, S100B has been viewed as a damage-associated molecular pattern (DAMP) involved in the inflammatory response, and serves as a generic receptor for the advanced glycation end products (RAGE) activator in the context of the inflammatory response (Xiao, *et al.*, 2014; Uspenskaya, *et al.*, 2015; Cao, *et al.*, 2017). Increasing evidence has shown that S100B binding to RAGE can promote the release of inflammatory cytokines via the activation of NF-κB, JNK, PI3K ,and P38 MAPK (Bianchi, *et al.*, 2010, 2011). These signaling pathways were known to be involved in the regulation of SLE (Zhi-Chun, *et al.*, 2012; Shi, *et al.*, 2015). In addition, substantial evidence has showed that S100B is associated with pathological injury or clinical severity in a variety of autoimmune disease (Wang, *et al.*, 2013; Gomez-Tourino, *et al.*, 2015; Lapa, *et al.*, 2017). Within this context, we hypothesized that S100B might be involved in the development of SLE.

The gene encoding S100B is located on chromosome 21q22.2-q22.3 and consists of three exons and two introns. Recently, genetic association studies have indicated that S100B polymorphisms are related to human diseases, such as invasive aspergillosis, autism spectrum disorder, bipolar affectivedisorder, and dyslexia (Roche, *et al.*, 2007; Egger, *et al.*, 2014; Matsson, *et al.*, 2015; Dix, *et al.*, 2016). In addition, genetic variants in the S100B gene have been reported as significantly associated with the higher expression of S100B (Liu, *et al.*, 2005; Hohoff, *et al.*, 2010). Given the important roles that the abnormal expression of S100B plays in the development of autoimmune and inflammatory diseases (Hofmann, *et al.*, 1999; Hwang, *et al.*, 2011; Bechmann, *et al.*, 2013; Gomez-Tourino, *et al.*, 2015), we hypothesized that SNPs in the S100B gene may influence the expression of S100B and ultimately be involved in the etiology of SLE. To test this hypothesis, we selected three SNPs (rs9722, rs881827 and rs1051169) in the S100B gene and performed a case-control study to investigate the association of these SNPs with susceptibility to SLE in a Chinese population.

## Material and Methods

### Subjects

A total of 313 SLE patients (63 men and 250 women, average age 38.05 ± 12.93 years) were recruited from the Department of Dermatology, Affiliated Hospital of Youjiang Medical University for Nationalities, Guangxi, China between January 2013 and September 2016. The diagnosis of SLE was based on the 1997 revised American College of Rheumatology (ACR) SLE criteria (Hochberg, 1997). Their medical records were reviewed with particular attention to neuropsychiatric manifestations, which were ascertained by relevant specialists, employing laboratory and imaging investigations and were objectively documented. The 396 controls (98 men and 298 women, average age 39.48 ± 12.10 years) were matched to the patients on the basis of age and gender, and they were recruited from Health Medical Center of the hospital during the same period. According to the thorough clinical and laboratory evaluation, none of them was found to have any history of autoimmune disorders. Data about demographic and clinical features were collected from hospital records or by questionnaire, and were reviewed by experienced physicians. Written informed consent was obtained from all participants, and this study was approved by the research ethics committee of our hospital.

### DNA extraction

Blood samples from all subjects were collected in EDTA-containing tubes. Genomic DNA was isolated from peripheral blood mononuclear cells using a DNA extraction kit (QIAGEN, China) according to the manufacturer’s instructions and then stored at -70 °C for later use.

### Determination of S100B genotype

Primer probes were designed using Primer Express Software (version 3.0) and synthesized by Applied Biosystems (Foster City, CA). The primers used were as follows: rs9722: ACAACACGGCTGGAAAGCTCAG (forward), GATGGAGACG GCGAATGTGACT (reverse), TTTTTTTTTTTTTTTTTTGCCAAACCTTTCCTGTAA CAGAGA (extended); rs881827: TGTGTGTGGAAGTC CCTGTCTCA (forward), CCCTGCACTGTGGTTGTTC CTC (reverse), TTTTTTTTTTTTTTTTTTTTTTTTTTT TGTTGCTGAAGTAACTCTTGGGAAC(extended); rs1051169: TCACCTTCA GGGCAGCTGAGAA (forward), TGGAAGGGAGGGAGACAAGCAC(reverse), TTTTTTTTTTTTTTTTTTTTTTTTTTTTTTTTTTTTTT TCACAAGCTGAAGAAATCCGAACT(extended). SNP genotyping was performed by SBE-PCR. Amplifications were performed in a total volume of 20 μL, comprised of 3.0 mmol/L Mg^2+^, 0.3 mmol/L dNTP, 1 U HotStar*Taq* polymerase, 1 μL genomic DNA, 1 μL PCR primer and 1x GC-I buffer (Takara, Japan). The PCR conditions included an initial denaturation step at 94 °C for 20 s, followed by 35 cycles with 20 s of denaturation at 94 °C, 30 s of annealing at 59 °Cm and 1.5 min of elongation at 72 °C, followed by a final elongation step of 72 °C for 2 min. PCR products were digested with shrimp enzyme (Promega, Madison, WI) and excision enzyme (Epicentre, Madison WI). An ABI PRISM 3730XL analyzer (Applied Biosystems) was used to sequence the PCR products, and GeneMapper4.1 was used to analyze the sequencing data. The samples were reanalyzed and verified by DNA sequencing when conflicting results occurred. In addition, approximately 10% of all samples were randomly selected to be confirmed by DNA sequencing, and the results were 100% consistent.

### Serum S100B determination

Serum samples from SLE patients and controls were separated from peripheral venous blood at room temperature and stored at -70°C until use. The quantity determination of serum S100B were performed by ELISA kits (No: RD192090100R, BioVendor-Laboratorní medicína) following the manufacturer’s protocol. The concentration of serum S100B was determined using a standard curve constructed with the kit’s standards over the range of 10-320 pg/mL.

### Statistical analysis

All data were analyzed by the SPSS software version 17.0 (SPSS, Inc, Chicago, IL, USA). Hardy-Weinberg equilibrium (HWE) was tested by the chi-square test. Demographic and clinical data between groups were compared by chi-square test or Student’s *t*-test. Logistic regression was used to estimate odds ratio (OR) and 95% confidence interval (95% CI). False discovery rate (FDR) approach was used to correct for multiple testing. In brief, the stringent *p*-value was considered statistically significant if it was less than 0.05. The haplotype analysis was performed by online SHEsis software (Shi and He, 2005), and *p* < 0.05 was considered to be statistically significant.

## Results

### Clinical characteristics of the study participants

A total of 313 SLE patients and 396 control subjects were included in this study. The clinical characteristics of the study participants are listed in Table 1. There were no significant differences in age and gender distribution between the case and control groups (*p* > 0.05).

**Table 1:** Clinical characteristics of the subjects.

Characteristics	SLE patients (n=313)	Controls (n=396)	*p-*value
Age, year (mean ± SD)	38.05 ± 12.93	39.48 ± 12.10	0.131
Gender (M/F)	63/250	98/298	0.145
Cutaneous vasculitis (%)	146 (46.65)	-	-
Family history of SLE (%)	89 (28.43)	-	-
Arthritis (%)	139 (44.41)	-	-
Pleuritis (%)	42 (13.42)	-	-
Malar rash (%)	93 (29.71)	-	-
Renal disorder (%)	161 (51.44)	-	-
Neurologic disorder (%)	71 (22.68)	-	-
Anti-dsDNA antibody-positive (%)	153 (48.88)	-	-
Anti-Sm-positive (%)	123 (39.30)	-	-
Anti-RNP-positive (%)	135 (43.13)	-	-
ANA positive (%)	286 (91.37)		-
Low levels of C3 (%)	127 (40.57)	-	-
Low levels of C4 (%)	101 (32.27)	-	-

SLE, systemic lupus erythematosus; Anti-dsDNA, double-stranded DNA antibody; Anti-Sm, smith antibody; Anti-RNP, ribonucleoprotein antibody; ANA, antinuclear antibody; C3, complement 3; C4, complement 4.

### Association of S100B polymorphisms with SLE risk

The distributions of the S100B gene rs9722, rs881827 and rs1051169 in SLE patients and controls are shown in Table 2. The genotype distribution of the three SNPs in the control group was in agreement with HWE (both *p* > 0.05). The minor C allele of rs1051169, relative to the major G allele, appeared to have a significantly increased risk of SLE (C vs. G: adjusted OR=1.46, 95% CI, 1.18-1.80, *p*=0.001). Similarly, a statistical significance was also found for the rs1051169 CC genotype, dominant and recessive model (CC vs. GG: adjusted OR=1.99, 95% CI, 1.32-3.02, *p*=0.001; CC+GC vs. GG: adjusted OR=1.54, 95% CI, 1.13-2.11, *p*=0.007; CC vs. GC+GG: adjusted OR=1.67, 95% CI, 1.16-2.42, *p*=0.006). The *p*-values remained significant after correction for multiple testing. However, no significant association between other SNPs (rs9722 and rs881827) and SLE risk was observed (*p* > 0.05).

**Table 2:** Association between S100B polymorphisms and risk of SLE.

SNPs	Controls (n=396)	SLE(n=313)	Adjusted OR (95% CI)*	P*	*P*_*BH*_
rs9722					
GG	182 (46.0)	154 (49.2)	1.0 (Ref)	-	
GA	170 (42.9)	134 (42.8)	0.93 (0.68-1.27)	0.646	0.745
AA	44 (11.1)	25 (8.0)	0.68 (0.40-1.16)	0.160	0.394
G	534 (67.4)	442 (70.6)	1.0 (Ref)	-	
A	258 (32.6)	184 (29.4)	0.87 (0.69-1.09)	0.211	0.396
Dominant (AA+GA vs. GG)			0.88 (0.65-1.18)	0.394	0.537
Recessive (AA vs. GA+GG)			0.70 (0.42-1.18)	0.184	0.394
rs881827					
GG	157 (39.6)	127 (40.5)	1.0 (Ref)	-	
GA	171 (43.2)	142 (45.4)	1.04 (0.75-1.44)	0.813	0.871
AA	68 (17.2)	44 (14.1)	0.82 (0.53-1.29)	0.389	0.537
G	485 (61.2)	396 (63.3)	1.0 (Ref)	-	
A	307 (38.8)	230 (36.7)	0.93 (0.75-1.16)	0.517	0.646
Dominant (AA+GA vs. GG)			0.98 (0.72-1.33)	0.888	0.888
Recessive (AA vs. GA+GG)			0.81 (0.53-1.22)	0.303	0.505
rs1051169					
GG	158 (39.9)	95 (30.4)	1.0 (Ref)	-	
GC	170 (42.9)	139 (44.4)	1.37 (0.97-1.92)	0.072	0.216
CC	68 (17.2)	79 (25.2)	**1.99 (1.32-3.02)**	**0.001**	**0.015** ^*^
G	486 (61.4)	329 (52.6)	1.0 (Ref)	**-**	
C	306 (38.6)	297 (47.4)	**1.46 (1.18-1.80)**	**0.001**	**0.015** ^*^
Dominant (CC+GC vs. GG)			**1.54 (1.13-2.11)**	**0.007**	**0.026** ^*^
Recessive (CC vs. GC+GG)			**1.67 (1.16-2.42)**	**0.006**	**0.026** ^*^

Boldface indicates significantly different. Ref: reference; OR: odds ratio; 95% CI: 95% confidence interval. *: adjusted by age and gender. *P*_BH_: *P* values corrected by Benjamin-Hochberg (B-H) method. *P*_BH_^*^ remains significant after FDR test is applied.

### Analysis of haplotype distribution between SLE patients and controls

The haplotypes frequencies of the three SNPs in S100B gene among the cases and controls were also estimated in our study. It was performed online using the SHEsis software, and the possible eight haplotypes are listed in Table 3. G-G-G and G-G-C were the two major haplotypes, accounting for 27.7% and 20.3%, and 30.2% and 14.5% in both SLE patients and controls, respectively. Moreover, we found the G-G-C haplotype to be associated with an increased risk of SLE compared with controls (OR=1.50, 95% CI, 1.14-1.98, *p*=0.004).

**Table 3:** Haplotype distribution in the patients with SLE and the controls.

Haplotype	Controls (2n=792)	SLE patients (2n=626)	OR (95% CI)	*p*
A A C	57 (7.2)	49 (7.9)	1.11 (0.75-1.65)	0.608
A A G	71 (8.9)	39 (6.2)	0.68 (0.45-1.02)	0.060
A G C	72 (9.1)	63 (10.0)	1.12 (0.78-1. 59)	0.545
A G G	59 (7.4)	33 (5.2)	0.69 (0.44-1.07)	0.098
G A C	62 (7.8)	57 (9.2)	1.19 (0.82-1.73)	0.370
G A G	117 (14.8)	84 (13.4)	0.89 (0.66-1.21)	0.457
G G C	115 (14.5)	127 (20.3)	**1.50 (1.14-1.98)**	**0.004**
G G G	239 (30.2)	173 (27.7)	0.88 (0.70-1.11)	0.291

Boldface indicates significantly different. OR: odds ratio; 95% CI: 95% confidence interval.

### Association of rs1051169 polymorphisms with clinical features

We further performed a stratification analysis by comparing the distribution of genotype and allele frequencies in rs1051169 between positive and negative patients in 13 specific clinical features. Significant differences were observed between the rs1051169 polymorphism and neurologic disorder (*p*=0.013, *p*=0.003, respectively) (Table 4). In addition, patients in the case group were further divided into two groups, which were the neurologic disorder (ND) group and non-neurologic disorder (NND) group. When we further estimated the rs1051169 polymorphism and the risk of neurologic disorder, we found the rs1051169 C allele, GC genotype, CC genotype and dominant model to be associated with increased susceptibility to neurologic disorder in SLE patients (C vs. G: OR=1.78, 95% CI, 1.22-2.59, *p*=0.003; GC vs. GG: OR=2.33, 95% CI, 1.14-4.77, *p*=0.019; CC vs. GG: OR=3.02, 95% CI, 1.39-6.53, *p*=0.004; CC+GC vs. GG: OR=2.57, 95% CI=1.31-5.04, *p*=0.005) (Table 5).

**Table 4:** Association analysis of rs1051169 polymorphism with clinical features.

Variables	+/-	Genotypes			*p*	Allele		*p*
		GG	CG	CC		G	C	
Cutaneous vasculitis	+	52 (31.7)	75 (45.7)	37 (22.6)	0.516	179 (54.6)	149 (45.4)	0.289
	-	43 (28.9)	64 (43.0)	42 (28.2)		150 (50.3)	148 (49.7)	
Family history of SLE	+	25 (28.1)	36 (40.4)	28 (31.5)	0.279	86 (48.3)	92 (51.7)	0.180
	-	70 (31.3)	103 (46.0)	51 (22.7)		243 (54.2)	205 (45.8)	
Arthritis	+	47 (33.8)	62 (44.6)	30 (21.6)	0.314	156 (56.1)	122 (43.9)	0.111
	-	48 (27.6)	77 (44.2)	49 (28.2)		173 (49.7)	175 (50.3)	
Pleuritis	+	15 (35.7)	20 (47.6)	7 (16.7)	0.371	50 (59.5)	34 (40.5)	0.169
	-	80 (29.5)	119 (43.9)	72 (26.6)		279 (51.5)	263 (48.5)	
Malar rash	+	31 (33.3)	42 (45.2)	20 (21.5)	0.567	104 (55.9)	82 (44.1)	0.274
	-	64 (29.1)	97 (44.1)	59 (26.8)		225 (51.1)	215 (48.9)	
Renal disorder	+	48 (29.8)	78 (48.4)	35 (21.7)	0.239	174 (54.0)	148 (46.0)	0.445
	-	47 (30.9)	61 (40.1)	44 (28.9)		155 (51.0)	149 (49.0)	
Neurologic disorder	+	12 (16.9)	35 (49.3)	24 (33.8)	**0.013**	59 (41.5)	83 (58.5)	**0.003**
	-	83 (34.3)	104 (43.0)	55 (22.7)		270 (55.8)	214(44.2)	
Anti-dsDNA	+	50 (32.7)	61 (39.9)	42 (27.5)	0.286	161 (52.6)	145 (47.4)	0.977
	-	45 (28.1)	78 (48.8)	37 (23.1)		168 (52.5)	152 (47.5)	
Anti-Sm	+	30 (24.4)	62 (50.4)	31 (25.2)	0.134	122 (49.6)	124 (50.4)	0.232
	-	65 (34.2)	77 (40.5)	48 (25.3)		207 (54.5)	173 (45.5)	
Anti-RNP	+	46 (34.1)	62 (45.9)	27 (20.0)	0.331	154 (57.0)	116 (43.0)	0.142
	-	52 (29.2)	78 (43.8)	48 (27.0)		182 (51.1)	174 (48.9)	
ANA	+	88 (30.8)	130 (45.5)	68 (23.8)	0.148	306 (53.5)	266 (46.5)	0.125
	-	7 (25.9)	9 (33.3)	11 (40.7)		23 (42.6)	31 (57.4)	
Low levels of C3	+	36 (28.3)	65 (51.2)	26 (20.5)	0.110	137 (53.9)	117 (46.1)	0.567
	-	59 (31.7)	74 (39.8)	53 (28.5)		192 (51.6)	180 (48.4)	
Low levels of C4	+	27 (26.7)	53 (52.5)	21 (20.8)	0.136	107 (53.0)	95 (47.0)	0.886
	-	68 (32.1)	86 (40.6)	58 (27.4)		222 (52.4)	202 (47.6)	

Boldface indicates significantly different. +: positive; -: negative.

**Table 5:** Association of rs1051169 polymorphism with neurologic disorder risk.

Group		+ (n=71)	- (n=242)	OR (95% CI)	*p*-value
Genotype	GG	12 (16.9)	83 (34.3)	1.0 (Ref)	-
	GC	35 (49.3)	104 (43.0)	**2.33 (1.14-4.77)**	**0.019**
	CC	24 (33.8)	55 (22.7)	**3.02 (1.39-6.53)**	**0.004**
Allele	G	59 (41.5)	270 (55.8)	1.0 (Ref)	-
	C	83 (58.5)	214 (44.2)	**1.78 (1.22-2.59)**	**0.003**
Dominant	GG	12 (16.9)	83 (34.3)	1.0 (Ref)	-
	GC+CC	59 (83.1)	159 (65.7)	**2.57 (1.31-5.04)**	**0.005**
Recessive	CC	24 (33.8)	55 (22.7)	1.0 (Ref)	-
	GG+GC	47 (66.2)	187 (77.3)	1.74 (0.98-3.09)	0.059

### Association between S100B polymorphisms and serum S100B levels

**Figure 1:**
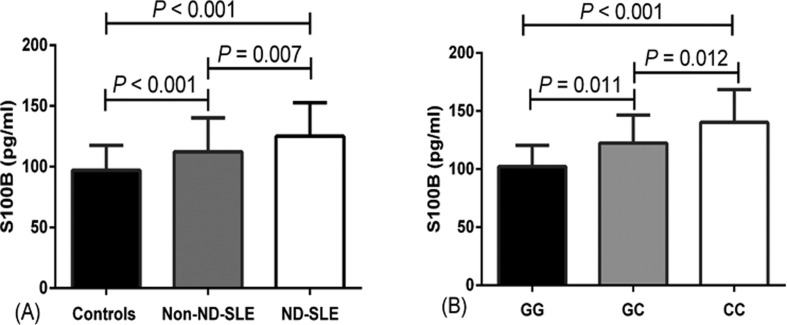
ELISA detection of S100B levels. (A) The SLE patients with neurologic disorder (125.08 ± 20.34 pg/mL, n=71) showed higher serum S100B levels than non-neurologic disorder patients (112.30 ± 27.98 pg/mL, n=71) and controls (97.16 ± 20.34 pg/mL, n=71), respectively [both *p* < 0.05]. (ND-neurologic disorder). (B) The SLE patients with neurologic disorder carrying the rs1051169 CC genotype (140.29 ± 28.17 pg/mL, n=24) showed significantly higher serum S100B levels than whose with GC (122.47 ± 24.11 pg/mL, n=35) and GG genotype (102.24 ± 18.26 pg/mL, n=12), respectively (both *p* < 0.05).

We also investigated the association between S100B polymorphisms and serum S100B levels. We found that the serum S100B levels in SLE patients with neurologic disorder were significantly higher than in non-neurologic disorder patients and controls (*p* < 0.05, respectively) (Figure 1A). Considering that the rs1051169 polymorphism may play an important role in the etiology of SLE, especially in the patients with neurologic disorder, we then performed a comparison between rs1051169 genotypes and serum S100B levels, and observed that the patients with neurologic disorder carrying the rs1051169 CC/GC genotypes presented higher serum S100B levels compared with those carrying the rs1051169 GG genotype (both *p* < 0.05) (Figure 1B).

## Discussion

In this study, we investigated the association between three SNPs in the S100B gene and SLE risk in a Chinese population. We found that the rs1051169 C allele, CC genotype, dominant model (CC+GC vs. GG) and recessive model (CC vs. GC+GG) were significantly associated with increased risk of SLE. Haplotype analysis showed that the G-G-C haplotype was associated with an increased risk of SLE. Moreover, further stratified analyses showed that SLE patients carrying the rs1051169 C allele, GC genotype, CC genotype and dominant model (CC+GC vs. GG) were more likely to develop neurologic disorder. In addition, we observed that in individuals carrying rs1051169 CC genotype there was an association with abnormal expression of S100B in SLE patients with neurologic disorder. Taken together, these findings indicate that the S100B gene rs1051169 polymorphism may play a critical role in the etiology of SLE, especially in patients with neurologic disorder.

The synonymous variant SNP rs1051169 is located in the promoter region of the S100B gene, and several studies have reported an association between the rs1051169 polymorphism and human diseases, however, the results were inconsistent. Zhai *et al.* (2011) found that the rs1051169 variant was correlated with schizophrenia patients’ poorer spatial ability in a Chinese population. Similarly, a case-control study conducted by Liu *et al.* (2005) reported that the rs1051169 GG genotype was associated with an increased risk of schizophrenia in a Chinese population. However, Guo *et al.* (2013) have tried to detect an association of the rs1051169 polymorphism with Parkinson’s disease in a Chinese population, but failed to obtain a positive result. In this study, our findings were in agreement with the positive results of Zhai *et al.* (2011) and Liu *et al.* (2005), as we found that the rs1051169 C allele, CC genotype, CC+GC vs. GG and CC vs. GC+GG had 1.46-fold, 1.99-fold, 1.54-fold and 1.67-fold increased risks of developing SLE, respectively. Furthermore, a stratified analysis showed that the rs1051169 C allele, GC genotype, CC genotype, and CC vs. GC+GG were associated with increased susceptibility to neurologic disorder in SLE patients. In addition, the SLE patients with neurologic disorder carrying CC or CG genotypes seem to exhibit relatively higher levels of expression S100B compared with those carrying the GG genotype. It is known that polymorphisms in the promoter region of certain genes might regulate their expression by altering the binding sites of transcription factors (Sun, *et al.*, 2007; Shao, *et al.*, 2017). We hypothesized that the synonymous SNP rs1051169 located in the exon of S100B may exert an influence on splicing, thereby affecting the levels of serum S100B, which ultimately potentiate S100B-mediated pro-inflammatory processes, increase SLE risk, and promote neurologic disorder development.

With regard to rs9722 and disease risk, contradictory results were also observed. Matsson *et al.* (2015) reported that the rs9722 T allele was associated with dyslexia in a German family. In a case-control study, Li *et al.* (2017) demonstrated that the rs9722 T allele was significantly associated with the risk of severehand, foot, and mouth disease. A positive result was also observed in schizophrenia patients (Zhai, *et al.*, 2011). However, Yang K *et al.* (2008) showed that the rs9722 polymorphism was not correlated with the risk of major depressive disorder in a Chinese population. Our results are in concordance with the negative result of Yang K *et al.* (2008). Several possibilities need to be taken into account to explain the negative results. Firstly, genetic polymorphisms play different roles in different diseases, especially in diverse ethnicities. Furthermore, we cannot rule out the possibility that the negative result is due to the relatively small number of subjects. Regarding the rs881827 polymorphism, up to now, a very limited number of studies has assessed the association of rs881827 polymorphism with human disease susceptibility. In our study, no association of rs881827 SNP with SLE risk was observed.

SLE is a complex chronic inflammatory disease. Although the exact mechanisms responsible for initiating SLE remain unclear, it is well known that inflammatory cytokines play an important and diverse role in the pathogenesis of SLE. S100B is a multigene family of small (-10 kDa) Ca^2+^ binding proteins, which can combine with RAGE to induce the secretion of a variety of thepro-inflammatory cytokines, such as TNF-α, IL-1β, IL-6, and IL-8 (Bianchi, *et al.*, 2010; Dang, *et al.*, 2014; Niven, *et al.*, 2015). Several studies have demonstrated that these inflammatory cytokines play crucial roles in the pathogenic process of SLE (Sun, *et al.*, 2000; Ye, *et al.*, 2014). Besides, previous study showed that serum TNF-α, IL-1β, IL-6, and IL-8 levels in central nervous system neuropsychiatric SLE (CNS-NPSLE) cases were higher than those in the control and non-CNS SLE groups (Wang, *et al.*, 2015). Moreover, higher serum S100B levels have been reported to reflect brain injury and increased permeability of the blood–brain barrier (BBB) (Yang XY *et al.*, 2008; Fragoso-Loyo, *et al.*, 2010). In our study, we observed that serum S100B levels in SLE patients with neurologic disorder were significantly higher than in non-neurologic disorder patients and controls. Based on this background, the positive results in our study were biologically reasonable.

Although the current study showed that S100B polymorphisms may play a critical role in the etiology of SLE, our study also has the following limitations. Firstly, the relatively small sample size may have limited the statistical power in our study. Secondly, all participants were recruited from the same hospital, so the possibility of selection bias cannot be ruled out. Thirdly, due to the lack of complete clinical data we cannot assess the impact of medical regimens on the results of this study. Another limitation is that data on environmental exposure is not available, which prevented further analysis of the effect of a gene–environment interaction on SLE risk. Therefore, further studies with larger sample sizes and including gene-environment interaction are warranted.

## Conclusions

Our results indicate that the S100B gene rs1051169 polymorphism may play a major role in the pathogenesis and development of SLE. Further studies with larger samples and in different populations are needed to confirm these findings.
